# AI Implementation Science for Social Issues: Pitfalls and Tips

**DOI:** 10.2188/jea.JE20210278

**Published:** 2022-04-05

**Authors:** Kota Takaoka

**Affiliations:** Artificial Intelligence Research Center, National Institute of Advanced Industrial Science and Technology, Artificial Intelligence Research Center, Tokyo, Japan

**Keywords:** child abuse, child maltreatment, AI, implementation, machine learning

## Abstract

The social implementation of knowledge and technologies that are effective in epidemiological and observational studies is essential for solving social issues. In particular, there have been few attempts to implement clinical practices and information communication technologies that utilize data in the field. We describe the four stages of social implementation: 1) redefining social issues as solvable problems, 2) finding technological solutions to solvable problems, 3) social implementation contributing to the solutions, and 4) horizontal deployment of effective methods for solving social issues. Introducing a use case of artificial intelligence (AI) social implementation in child-abuse response conducted by our team, we discuss pitfalls and tips as a frame of reference to demonstrate data utilization as social infrastructure for solving social issues and to consider practical solutions in a logical manner.

## BACKGROUND

In recent years, artificial intelligence (AI) has begun to be used to understand common diseases and their risk factors. For example, early detection of depression and recommendations for early improvement based on big data,^1^ suicide prevention systems using big data as part of the Durkheim Project promoted by the Defense Advanced Research Projects Agency,^2^ and surveillance for school gun-shootings^3^ are already underway. In the fields of public health and epidemiology, AI technology has been implemented in society for the purpose of early detection of abnormalities, search for reasons, prognosis prediction, and precision care using big data, and research has been conducted. At the same time, the latest technologies, such as AI, are beginning to be applied to social issues. Today, people encounter numerous social issues in their daily lives, such as poverty, lack of education, abuse, disability, inequality, and crime. If our society does not invest in long-term solutions, the risk of negative impact on society increases. Sustainable development goals (SDGs) for policymakers aim to solve such issues. For frontline practitioners, schemes are being established for various players to collaborate to bring about positive collective impacts. Environmental, social, and corporate governance (ESG) investment frameworks for investors, which aim to develop and accelerate projects that solve important social issues, are also beginning to be utilized worldwide. Technological innovation has emerged as a key driver of social problem-solving involving business communities. For example, AI and sophisticated computational algorithms, such as machine learning, were developed to meet the increasing accumulation of big data through Information and Communications Technology and Internet of Things (IoT) technologies in our daily lives. Today, AI has become intricately linked with our daily lives and activities, with some functions and services that have already become indispensable.

**Figure 1.**
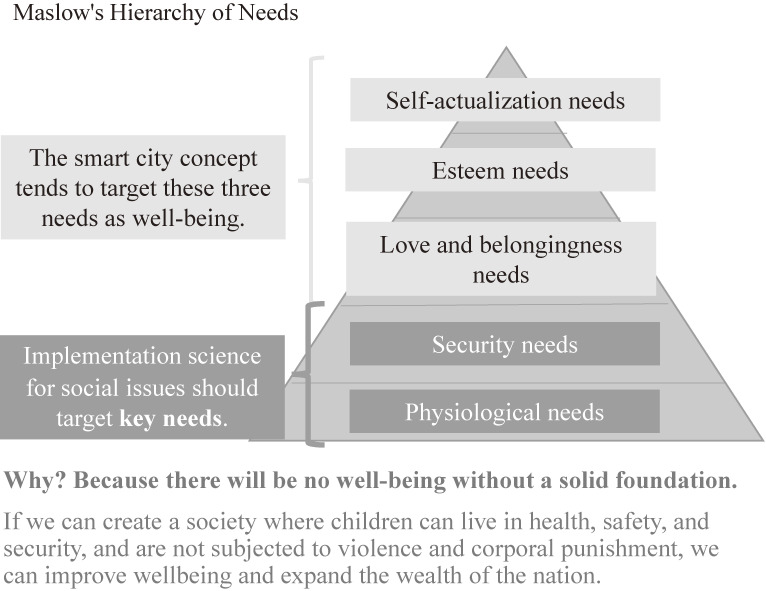
Targeted needs in Maslow’s hierarchical model

However, the question remains: how can technological innovation contribute to social issues? The key to this answer lies in the physiological and security needs of Maslow’s hierarchical model of human needs (see Figure 1). Recent development of information communication technology (ICT), starting with the IoT, has led to a digital transformation that has enabled the utilization of data and digitization of entire cities. The concept of smart cities aims to create a society in which the well-being of each individual is maximized and everyone is independent. Many ICT companies and governments, however, do not pay attention to the physiological and security needs that are essential for achieving this vision. As social issues are often concentrated in the two basic needs mentioned above, social implementation that utilizes data and AI to address these two needs is inevitably required. Considerable international attention has been paid to how AI and other technologies can be used in the areas of ethical, legal, and social issues (ELSI). However, very few advanced examples in the world of social implementation of AI in the areas exist. Therefore, social implementation is desirable in these areas to address social issues.

Typically, the traditional research-to-implementation sequence includes three steps: evaluation of efficacy in a laboratory environment or ideal research conditions; evaluation of effectiveness in the real world; and social implementation research.^4^ In recent years, protocols for social implementation, such as the Consolidated Framework for Implementation Research and the Theoretical Domains Framework for social implementation, have been developed.^5^ However, social implementation is not just a matter of applying research results, but also ensuring that they are easy to use in the field. Furthermore, social implementation should be effective in solving problems and be practical enough to accumulate knowledge and change the way people work.^6^ To achieve these goals, it is important to build consensus with stakeholders in the field and design social implementation that can repeat the loop of practice and evaluation using data^7^ (ie, how to collaborate with the stakeholder in the field to “utilize data” as an appropriate solution). This paper aims to outline tips for researchers and potential pitfalls that they may encounter in social implementation research. In this paper, we define AI as the use of data, such as machine learning and probabilistic modeling, to solve social problems.

Some of the fields have responded to social issues involving a wide range of complex factors with the experience and senses of practitioners. The purpose of implementing AI for solving social issues is to find solutions logically and regularly using data, in addition to the perspective of such heuristic approaches. To achieve successful implementation, there are three main objectives of implementing AI in solving social issues. The first objective is to enable people to have a common understanding of complex cases. For this purpose, it is important to organize typological patterns as unsupervised learning for AI. For example, there is a clustering method that performs dimensional compression on the features of a case that consists of a variety of factors. As a result of clustering, typological patterns on common issues and backgrounds in the case can be used to promote common understanding among the stakeholders. The second purpose is to provide a means of predicting information about the future that is difficult for humans to determine and to consider how to deal with it, even in highly uncertain situations. In the initial response phase, information is often lacking, and practitioners need to make decisions under uncertainty. In such situations, AI predictions and inferences through data can be useful to consider because the future is difficult for humans to judge. The third objective is to support the best possible choices in the field from a management-level perspective. In the field, operations need to proceed smoothly within the limits of staffing, organization, and budget. Therefore, it is not always possible to take ideal measures, and it is important to make the best possible choices within the resources available. To support such practitioners, AI optimization methods are beneficial. AI can contribute to knowledge of how to balance the workload and human resources with the responses necessary to solve problems. In the following section, we discuss pitfalls and tips for AI social implementation research.

## PITFALLS AND TIPS

We describe four different stages of an essential scheme for AI social implementation to resolve social issues. In each stage, we introduce an example of a social implementation experiment conducted in the field of child abuse.

### Step 1: Re-define social issues as solvable problems

In this paper, we employ the case of AI implantation for responding to child abuse cases by the Child Guidance Centers in Japan as a use case. The main issues of child guidance centers in dealing with child abuse are thought to be improving the quality of decision making, enhancing operational efficiency, and professional training for practitioners. The most important point to be considered when dealing with child abuse is to prevent serious accidents from occurring and to avoid overlooking them. Once this point is ensured, measures must be taken to deal with the increasing number of reported child abuse cases. To achieve this, the priority is to use AI technology and information communication technology to contribute to improving the quality of decision-making and to support the prevention of serious accidents. Moreover, it is important to establish a system that is automatically linked to the improvement of operational efficiency and the development of human resources based on the ameliorated quality of decision-making. The reason for this is that child welfare work has different difficulties from general administration work. For example, in general administration work, an application form is given to a client and the client is asked to fill out the form correctly, on the assumption that the client has provided correct information. On the other hand, at the Child Guidance Center, the staff needs to judge ambiguous information about reported families and children. In the context of child abuse cases, caregivers may say “my kid fell on the slide” even though the trauma was caused by the guardian hitting the child. Believing what the guardian says can lead to serious accidents. Practitioners may have a normalcy bias, which leads them to believe that “it’s probably okay” when they are unsure of their decisions. By comparing the result of AI simulation with the practitioners’ decision-making, they can calmly adjust their own bias. Even practitioners who have been working with a heuristic approach can use AI using data to back up their experience and senses. AI will become an assistant for practitioners in the field and lead to an improvement in the quality of their decisions.

#### Differences between AI implementation and traditional epidemiological research

The implementation of AI and conventional epidemiological research have in common the fact that they set the desired outcomes and examine them based on high-quality data. The difference is that epidemiology deals with the characteristics of the population by observing the characteristics of each group and evaluating the effects of interventions for the group, while AI enables prediction on an individual basis. Therefore, AI can be useful to build a precision-care approach that suits the characteristics of individuals. However, when we conduct the precision-care approach, AI implementation should always consider ethical issues. Particularly in interpersonal and community support with AI, there can be implicit discrimination and favoritism towards certain individuals due to sampling size, bias effects, and tuning effects of AI. Political correctness and social norms must be carefully considered in AI implementation design.

#### Set the outcome of the social problem to be solved

In social implementation, as in epidemiology and effectiveness research, setting outcomes is of utmost importance. For the field, ascertaining users’ needs and developing schemes to meet these needs is most essential.^8^ Outcomes should be carefully set, while staying close to the field and achieving consensus on which issues should be solved first. In the foundation of the implementation design, it is necessary to reserve enough time and effort to achieve the desired outcome.

#### Design and preparation for social implementation

To solve these issues, the most important design aspects for social implementation is how the user’s work will change before and after social implementation and how this will solve issues.^9^ When users employ services in which AI and other technologies are implemented after studying the workflow, it is important to design the data flow as a system architecture and to determine detailed operations of the ideal business after implementation. Here, it is necessary to set up a hypothesis of what the issues are and to explain them in an easy-to-understand manner. When it comes to large-scale social implementation, researchers must assume the following three perspectives: 1) the perspective of the issue itself (to draw the story of the issue solution); 2) users’ perspectives, such as those of field professionals (to obtain a bird’s eye view of the issue); and 3) managers and the municipalities’ perspectives (to manage the operations and make various political adjustments). It is crucial for researchers to envision these three perspectives in advance.

#### Building relationships with the field

In areas where people are not accustomed to using data, there is a history of dealing with difficult cases based on their experience and understanding, creating a sense of pride in the field. Therefore, even if a system for referencing data is proposed, dissatisfaction with the cost of changing work roles and opposition to implementing a new system due to psychological reactance can naturally occur. Especially in busy sites, learning something new may be interpreted as an increase in their workload; anger or confusion may be expressed as to why they should cooperate with the research. Thus, it is necessary to explain to the entire team, in advance, that such reactions may be encountered in the field and to ask participants for their understanding and cooperation. To overcome such situations, it is invaluable to build consensus by explaining to them that the purpose of social implementation is to solve a problem that is common to both the researchers and the field generally. This is not only a technical issue, such as a matter of negotiation skills; it is also important for researchers to show leadership with a mission.

#### Use-case example: Organizing issues at child guidance centers that respond to child abuse

**Figure 2.**
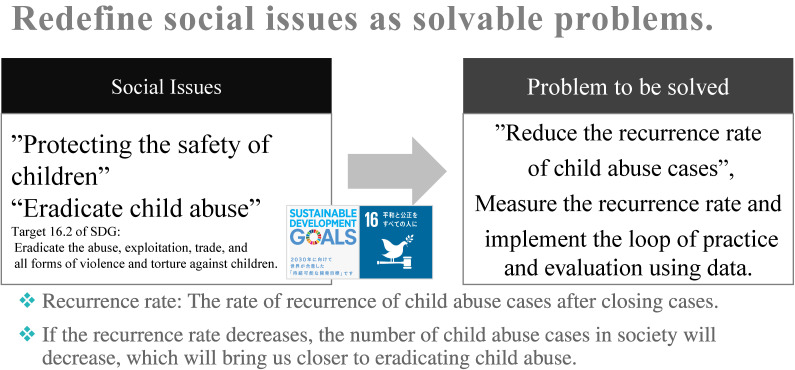
Redefining the social problem of child abuse into a solvable problem

Protecting children and ensuring their safety in the context of child abuse is a social issue that falls under SDG 16.2. In 2012 in Japan, there were two cases of child abuse deaths at one of the child guidance centers in a prefecture, leading to both local practitioners and the governor demanding change in the verification of cases of child abuse deaths to evidence-informed practice. In 2013, after receiving consent for data collection from both parties, the author began collecting digital data on risk assessment items at the time of the report. Even with a small sample, the data were analyzed, and the results were repeatedly fed back to the centers every 3–6 months. We found that different child guidance centers may have different protection standards with similar risks. This was relayed to the directors of the Child Guidance Centers in the prefecture, who formulated a new work policy in the field: “If the safety of a child is in doubt, protect the child.” Since then, we have continued to accumulate data and monitor our work regularly, monitoring the outcomes every year. One of the outcomes was the “reduction in the recurrence rate of abuse.” The organizational goal is to prevent the recurrence of child abuse cases handled by the child guidance center. If the recurrence rate decreases, the number of child abuse cases as a whole will decrease, implying that our society is closer to eliminating child abuse. Based on this perspective, we built a consensus in the field and redefined the problem by focusing on the recurrence rate (see Figure 2). After obtaining consensus from the field to set the recurrence rate as an outcome, we used prospectively collected data from 2014 to the present to predict the recurrence rate using machine learning and infer what actions should be taken in the field to reduce the recurrence rate using probability modelling. As a result, it became clear that providing temporary protection to high-risk abuse cases in the initial response phase would contribute to reducing the recurrence rate. It was also agreed that it would be good if the results could be referred to in real-time (eg, at the site of home visits), and the trial of AI implementation began.

### Step 2: Finding technical solutions to solvable problems

In this section, we summarize how to collect, evaluate, and utilize data for a problem to be solved as a preliminary step in implementing AI. Because there are many published materials on AI algorithms, we focus on data collection design and data handling in this section.

#### Creating, accumulating, and analyzing data from field operations

When the problem to be solved is set by the field and the researcher, it is necessary to consider whether the existing data are sufficient or whether there is a need to collect new data. This is because social implementation needs to answer for whom and how such data are useful.^10^ Especially in the field of social work, there are many cases where business records are kept on paper and are not digitized. In some cases, even if they are digitized, the database is not standardized and thus cannot be used for research. However, lack of data is a common occurrence in social implementation research, and there is merit in being able to discuss what kind of data would be useful according to the workflow in the field. Good social implementation always requires a set of good data.

In addition, domain knowledge of the field is of the utmost importance for collecting labels for supervised learning. The labelled data are necessary for an AI to learn from the data of correct answers provided in the field in advance. For example, in the case of survival analysis, if you want to know if patients are still alive after 5 years, data on death and survival will be the labels for supervised learning. Therefore, it is imperative to design system architecture that includes a database in which business records in the field can be directly used as labeled data.

Furthermore, standardized data should be collected using reliable and validated scales. However, this is insufficient, because even if standardized procedures are prepared for research, high-quality data cannot be obtained without training and briefing sessions for field staff to master them.^11^ Therefore, in social implementation, it is important to adopt the “research as service” perspective because acquiring high-quality data for the purpose of conducting research is linked with practical implementation processes in the field.

#### Sampling for imbalanced data

Depending on the problem to be solved, the balance between positive and negative outcomes may be disturbed due to the prevalence of other factors. For example, if we consider child abuse deaths as an outcome, less than 1% of all abuse cases will be positive. In the case of imbalanced data, adjustments must be made in the sampling stage. There are several methods to achieve this, but one of the most popular in recent years is the synthetic minority oversampling technique (SMOTE). According to Chawla et al,^12^ “SMOTE provides more relevant minority class samples to learn from, thus allowing a learner to carve broader decision regions, leading to more coverage of the minority class.” If you have imbalanced data, using methods such as SMOTE must be considered at the sampling stage.

#### Feature extraction considering sparse

The most effective method of building AI is to obtain a large sample size. However, administrative databases sometimes have too many items, including items that are irrelevant to their essence. If AI required all the data to get the output result, the practitioners must bear the burden of tremendous data input. It may lead to missing input values or data fragmentation because of their workload. For this reason, sparse modelling is useful in searching for the minimum number of features required and reduces the workload for field staff by allowing them to preferentially investigate and input data for the selected features in the field. Thus, to improve the performance of the model, it is important to design for efficiency based on the workflow in the field. By focusing on the most important features, the model is simplified, making it easier for people to understand the background that the model explains. At the same time, selecting features that correspond theoretically and rationally to real-world phenomena through domain knowledge will also improve the interpretability of the prediction results. It can also reduce the computational cost and training time, which is useful for feature extraction when using machine learning and Bayesian statistics.

#### The contribution of eXplainable artificial intelligence (XAI)

Solving social issues is often relevant for judiciary and governmental authorities because it often requires legal intervention. In other words, administrative officials may be likely to become stakeholders of the social implementation research, which inevitably makes them accountable for all their actions and decisions. While machine learning and deep learning algorithms are good at predictions and can be reproduced, it is very difficult to ensure explainability. Thus, the adoption of eXplainable AI (XAI) algorithms has been garnering attention globally as a means to utilize data to solve social problems and make policy decisions for administrative officials.^13^ The details of XAI are beyond the scope of this paper, but they include the importance of traditional methods, such as linear regression, logistic regression, and generalized linear modeling, as well as the contributions of machine learning algorithms, such as decision trees, naïve Bayes, and k-nearest neighbor methods. Currently, other methods, such as statistical causal inference, local interpretable model-agnostic explanations (LIME),^14^ and Shapley additive explanations (SHAP),^15^ should also be considered.

#### Evaluation based on PR and ROC curves

In epidemiology, classification prediction is often performed using an evaluation index based on the receiver operator characteristic (ROC) curve and precision recall (PR) curve. The ROC curve takes the false positive rate on the horizontal axis and the true positive rate (recall) on the vertical axis. This allows us to determine the accuracy of the prediction over the entire ranking of the ROC curve. In contrast, the PR curve takes the recall rate on the horizontal axis and the precision rate on the vertical axis. Especially when there are a lot of negative data in an imbalanced dataset, it is desirable to refer to both the ROC curve and the PR curve.

#### Use-case example: Implementing technology to solve problems in child guidance centers deal with child abuse cases

**Figure 3.**
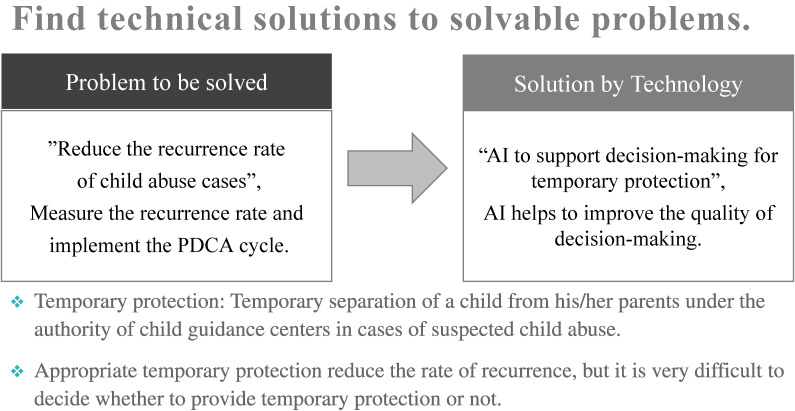
Technical solutions to solvable problems

In child abuse counseling services, the most important point is to prevent serious accidents from occurring or being overlooked. From 2014 to 2018, we collected data on more than 6,000 cases of child-abuse response in a municipality, including demographic factors, risk assessment data, whether the child was temporarily protected, and whether the child abuse recurred. We used gradient boosting, a machine learning algorithm, to predict whether temporary child protection should be provided. At the same time, we implemented a Bayesian network on a cloud server to enable real-time probabilistic causal inference in the field on how the prognosis of recurrence rates would change with and without child temporary protection.^16^ To ensure the security of personal information, we built a closed network that connects directly from users’ tablets to the cloud without connecting to the Internet, and developed an AI decision support system, the assistant of intelligence for child abuse and neglect (AiCAN), that allows child guidance center staff to bring their tablets to visit sites and enter and view risks and business records (See Figure 3).^17^

### Step 3: Social implementation based on the solution

In this section, we describe the implementation stage and summarize the key points to specify how the system should be used in the field work for social implementation.

#### Preparing operations manuals for implementation

In the social implementation phase, it is important to explain the concrete operations, such as who, when, where, to whom, what, why, and how to use the implemented new system, including products and apps. For this purpose, it is essential to design procedures and prepare manuals in which field users, researchers, and developers can systematically work on social implementation and trials in a language that is common to some extent.^9^ For instance, it is helpful to start with the assumption of the user’s personas who will use the system. The person responsible for implementation should specify in advance whether the users are patients/clients, support staff, or managers, and then clearly communicate what and how they would like the specific procedures to change in their past and future work. What is particularly important is that the researcher should actively grasp the detailed problems and unique issues each time, observing what the stakeholders in the field want to change and the user’s motivation for social implementation in the field.

#### Effectiveness evaluation and agile trial methodology

There are two main directions for evaluation through social implementation. The first is to measure the degree of achievement of outcomes that have been assumed in advance. This method compares the effects before and after implementation using quantitative indicators. The second is the evaluation of the research as a service, which aims to refine the implemented services and products and collect users’ quantitative and qualitative feedback in a short trial period. In particular, the second direction requires an agile trial methodology for research and development, in which the research and development goals for social implementation are segmented, trials are conducted on a small scale, and the loop of research, development, improvement of the product, and implementation is completed in a short period of time. The cycle of retaining functions that worked well and improving those that did not is repeated quickly from research to social implementation; agile research and development is, thus, often suitable for social implementation.

#### Use-case example: Social implementation of AI at a child guidance center for dealing with child abuse

**Figure 4.**
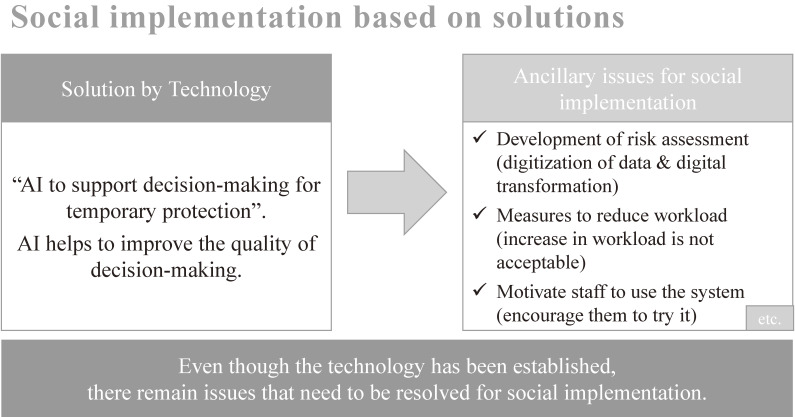
Social implementation based on solution method

The technology for predicting the need for temporary protection and inferring the future recurrence rate was based on the data of one municipality accumulated from 2014 to 2018. However, even though the technology was established, there were many issues that needed to be resolved prior to implementation. For example, we had to deal with various failures related to social implementation, such as digitization of risk assessment data, development of a system that contributes to reducing the workload so that it does not become a burden, and training and motivating staff to use the system. It was imperative to spare no daily effort to deal with the related ancillary matters (see Figure 4).

### Step 4: Expanding the effects of solving social issues

#### Horizontal expansion of effects and exit strategies

Once the effects of social implementation have been demonstrated, it is important to attempt the method at other sites and confirm its effectiveness through the same formative evaluation.^18^ If the method is effective, it is judged to be highly reproducible in solving the problem. Then, the next phase is to deploy it widely.^19^ The cost perspective is also important in widespread deployment.^20^ Therefore, once the research phase to confirm the effectiveness is over, the next step is to implement the effectiveness in more sites and to expand the social impact. When implementing a product in a society, researchers must always draw up an exit strategy. Examples of exit strategies include technology transfer to private companies after acquiring intellectual property or spinning out the research lab team and turning it into a start-up company. Additionally, if the budget and manpower for expansion are not available, open innovation through open science and creative commons can be conducted.

#### From a scientist-practitioner model to a sustainable service team as a research and development model

The demonstration experiments in social implementation are at the research stage to measure the effects; however, to develop the technology horizontally and implement it in society in earnest, we need to move to the commercial stage, where we can continue to create value sustainably. For this reason, social implementation does not end with research but requires the provision of sustainable services and the drawing up of expansion phases, including lateral expansion into other fields. Therefore, the traditional scientist-practitioner model for an individual in healthcare is insufficient for social implementation science. Rather, a team representing a research and development model that continues to provide a loop of clinical care, research, education, development, marketing, and sustainable service provision is essential. Consequently, we name it a “sustainable service team as a research and development” (SSTRD) model, which should be developed because collaboration between industry, academia, and government is important for social implementation research so that small projects can be created, nurtured, and expanded horizontally.

#### Use-case example: Horizontal deployment of AI in child guidance centers that respond to child abuse

**Figure 5.**
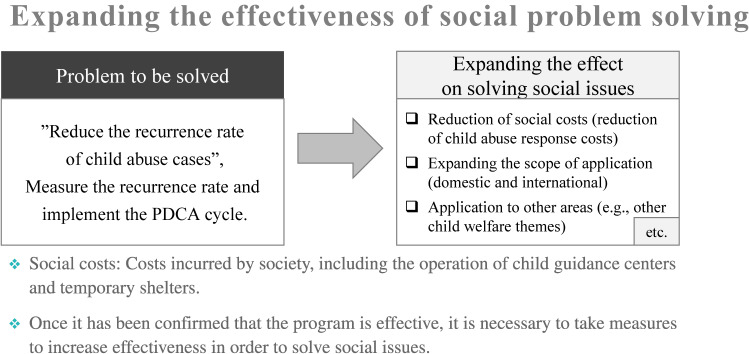
Strategies for increasing effectiveness of social problem solving

We are currently assessing the effects of these initiatives in the demonstration experiment. To expand the effects, it is necessary to develop strategies to reduce social costs, advance technologies that can be used not only in domestic but also in international countries in the near future, and collaborate with various stakeholders to expand the knowledge gained from child guidance centers to other institutes (eg, medical hospitals, law enforcement, child welfare agencies, schools, and nurseries) in order to deal with child abuse cases effectively and efficiently. Moreover, it is necessary to consider the value of social implementation to expand the effectiveness to other fields, such as domestic violence, sexual abuse, welfare, and bullying in schools (see Figure 5). To further solve the social issue of child abuse, it is necessary to take on the challenge of future development by maintaining the stance of a research institute responsible for the creation of new and highly innovative intellectual property, while leaving the perspective of providing services that sustainably solve problems in the field to the business side.

## FUTURE PROSPECTS

To date, electricity, gas, water, and other resources that support our daily lives have served as infrastructure, which is an important foundation for human activities in developing public health in a wide range of areas. However, when we consider that research results in epidemiology are important findings directly related to the infrastructure of human life, data contributing to child safety can also be considered as part of the nation’s social infrastructure. This is because we have already entered the age of an information society. It is necessary to share appropriate case information in a timely and prompt manner and to create a mechanism for social implementation so that AI and data can be available to make decisions in the field and solve social issues. This will lead to the creation of a future in which the safety of children is integrated into the social infrastructure. Social implementation will become social infrastructure when it becomes possible to routinely find logical solutions to problems using various types of data. In the future, it is expected that the results of various basic and clinical research projects will be developed into social implementations to solve social issues.
